# Clinical consequence of the 4-week X-ray control after ESIN osteosynthesis of forearm fractures in children

**DOI:** 10.1007/s00068-024-02721-6

**Published:** 2025-01-15

**Authors:** Erik Wegner, Nora Laubach, Phillip Schippers, Lotte Schierjott, Rujuta Shrotriya, Erol Gercek, Philipp Drees, Sven-Oliver Dietz

**Affiliations:** Department of Orthopaedics and Traumatology, University Medical Center of the Johannes Gutenberg University, 55131 Mainz, Germany

**Keywords:** Forearm fracture, Pediatric, ESIN, X-ray, Radiation exposure

## Abstract

**Purpose:**

The absence of evidence based general guidelines for radiographic follow-ups of pediatric diaphyseal forearm fractures treated with ESIN results in an arbitrary array of X-ray examinations. In most pediatric traumatology departments, an X-ray check is carried out 4 weeks after ESIN osteosynthesis of forearm shaft fractures to detect incipient consolidation of the fracture. However, the elevated sensitivity to ionizing radiation requires special precautions in the pediatric populations. To prevent the indiscriminate use of this diagnostic tool it is necessary to assess the consequence of routinely taken X-rays at the 4-week follow-up.

**Methods:**

This retrospective analysis included 219 pediatric patients who underwent ESIN treatment for a diaphyseal forearm fracture between 2010 and 2018 at the same pediatric trauma center. The primary outcome was defined as the prevalence of aberrant radiographic findings (ARF) during the 4-week follow-up with an otherwise normal clinical course and examination. Pathologies in the physical exam and irregularities in the medical history were summarized as non-radiological abnormalities (nRD). Binary logistic regression was calculated to identify risk factors associated with an increased likelihood of an ARF.

**Results:**

Of the 219 pediatric patients included, 123 patients had no nRDs at the 4-week follow-up. Only one patient was found to have an ARF (absolute risk = 0.008). Regression showed that the odds of receiving an ARF increased significantly as other nRDs were detected (p = 0.012, OR 14.554). A positive correlation was found especially for irregularities in the medical history (n = 59, p = 0.003, OR = 8.134).

**Conclusion:**

This study provides evidence that standardized radiographic follow-up 4 weeks after surgical treatment has no clinical consequences if the course of the fracture is otherwise uneventful. It should be strictly reserved for pediatric patients with a complicated course.

## Introduction

The forearm accounts for more than a third of all pediatric fractures, of which one in five occurs in the diaphysis. Especially both-bone forearm shaft fractures cause significant morbidity in this age group [1]. Elastic stable intramedullary nailing (ESIN) represents a commonly used treatment option for pediatric forearm shaft fractures requiring surgical care [2]. Its minimally invasive nature, low complication rates and its superior functional results with dispensable cast immobilization have led to a gradual shift away from conservative therapy towards surgical stabilization in recent decades [3–5]. In the absence of evidence-based guidelines, postoperative management is left to the practitioner. For this reason, children are X-rayed with varying frequency postoperatively [6]. Indiscriminate use of this diagnostic tool, however, should be avoided in the pediatric population, as their tissues are highly susceptible to the oncogenic properties of ionizing radiation [7, 8]. Although the radiation exposure from a single conventional X-ray is comparatively low, the frequency of its use contributes significantly to an increased collective radiation risk. Furthermore, repeated examinations may result in high cumulative doses and may also markedly raise the individual radiation risk [8, 9]. In addition to the assessment of medical necessity, the economic viability of routinely carried out postoperative X-ray follow-ups should also be scrutinized in times of escalating healthcare costs [10, 11].

The primary aim of this study was to determine whether there is a clinical need for a radiological follow-up 4 weeks postoperatively with an otherwise normal course of the fracture treatment in order to evaluate the clinical and economic benefit of this examination. To this end, the clinical and radiological data of 219 children with 220 forearm shaft fractures and ESIN osteosynthesis were retrospectively analyzed.

## Methods

### Study design and patient selection

For this retrospective, single-center study, pediatric patients were recruited between April 2010 and November 2018 at the Department of Orthopaedics and Trauma Surgery of the University Medical Center in Mainz, Germany. All children aged 1–14 years with a radiographically confirmed unstable forearm shaft fracture with exclusive ESIN osteosynthesis of both bones and a fully documented follow-up period to implant removal at the same level one pediatric trauma center were included. The follow-up at week 4 had to include a standardized two-plane radiograph of the affected arm.

**Fig. 1:**
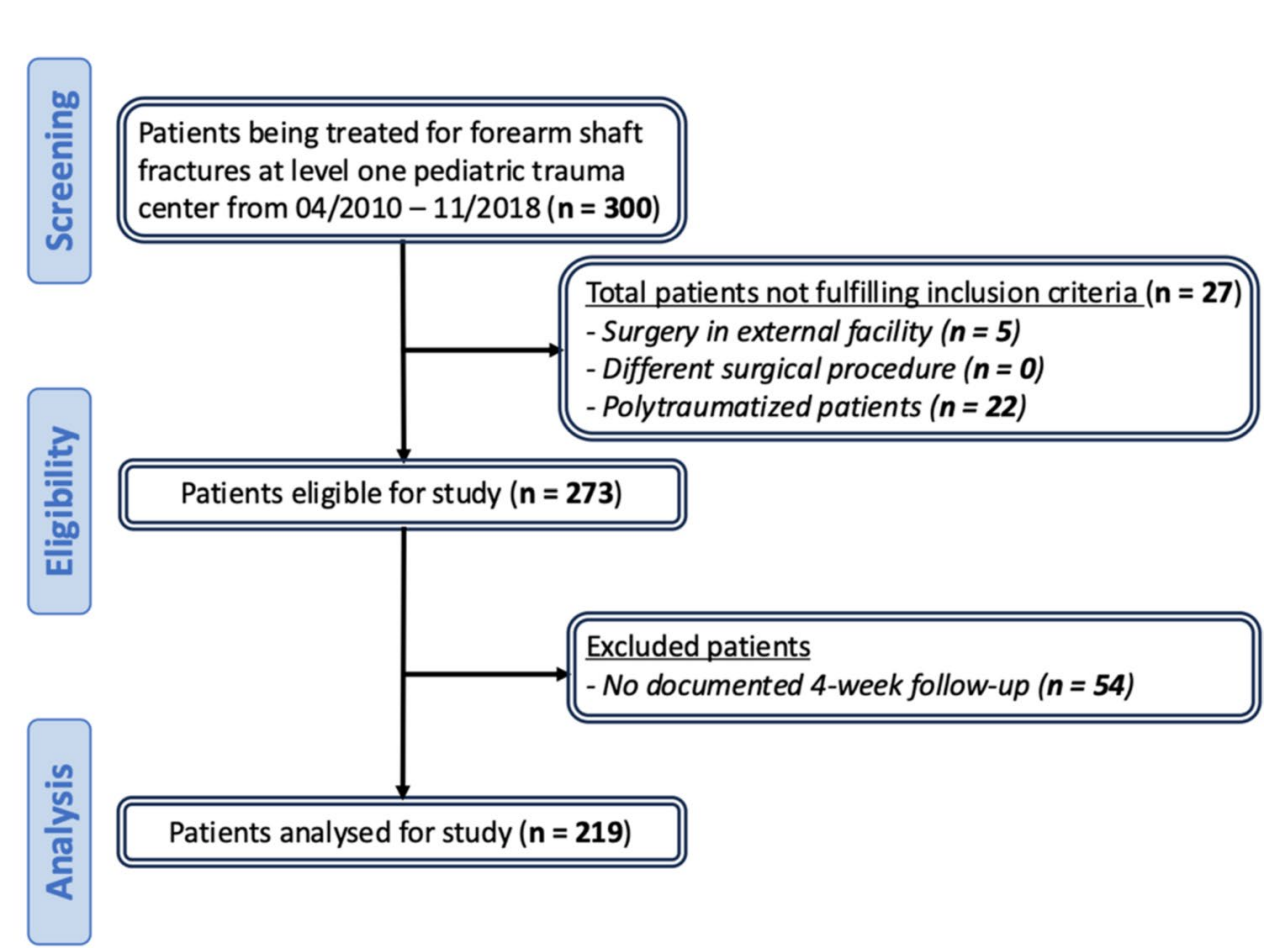
Patient selection flow chart

Out of a total of 300 children treated for forearm shaft fractures at our center, 27 patients were excluded because they had either been treated surgically at another center or had suffered the injury as part of a polytrauma. Because clinical and radiological follow-up at 4 weeks was not performed at our center, a further 54 patients were excluded. This left 219 patients available for analysis (Fig. 1).

This study was conducted in accordance with the Helsinki Declaration and approved by the ethics committee Rhineland-Palatinate, Germany (2020–14844-retrospektiv).

### Surgical technique

The surgical technique follows the general recommendations for ESIN osteosynthesis in forearm shaft fractures. Both bones had to be stabilized with an ESIN (Titanium Implants, Depuy Synthes^®^, Umkirch, Germany) each. In case of unsuccessful closed reduction, conversion to an open reduction technique was performed [6, 12, 13]. The procedure was not limited to one person, but could be performed by any orthopedic specialist at the clinic.

### Post-operative protocol

**Fig. 2:**
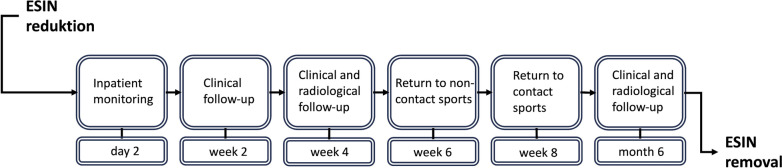
Post-operative protocol following ESIN osteosynthesis after both bone forearm fractures. A radiographic follow-up is routinely performed to verify bony consolidation and reduction 4 weeks after surgery

**Fig. 3:**
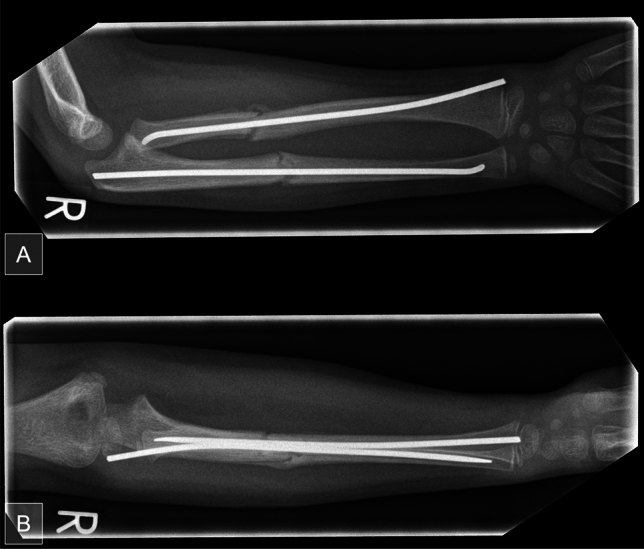
Exemplary image of a radiographic follow-up in an 8-year-old girl 4 weeks after ESIN osteosynthesis. Callus formation is found in time with anatomical reduction **A** in a.p and **B** in lateral view

Postoperatively, patients were discharged on the second or third postoperative day. Cast immobilization was avoided to facilitate immediate return to movement. The first clinical and radiological follow-up was carried out after 28 days. Sufficient callus formation allowed for return to non-contact sports at the end of postoperative week 6 followed by contact sports at the end of week 8. Full implant removal was performed after radiographic confirmation of a completed bony remodeling 6 months after surgery. Any changes to the procedure as depicted in Fig. 2 were defined as a deviation from the standard post-operative protocol. A summary of the deviations is shown in Table 1 (Fig. 3).

**Table 1:** Devations from the post-operative protocol

Deviation
Additional physical exams or X-rays
Additional wound management
Long arm casting
Re-osteosynthesis
Antibiotic therapy
Early implant removal
Prescription of physiotherapy
Referral to a specialist center

### Data analysis

Statistical analysis was performed in cooperation with the Institute of Medical Biostatistics, Epidemiology and Informatics (IMBEI) of the University Medical Center in Mainz. The statistical software IBM SPSS Statistics 29 (IBM, Arnonk, NY, USA) was used for evaluation and visualization. To identify risk factors associated with an increased likelihood of an abnormal radiograph or even a change in postoperative procedure, a binary logistic regression was calculated. The basic descriptive data were presented as median and interquartile range (IQR). The significance threshold was defined as p ≤ 0.05.

## Results

### Clinical characteristics of pediatric patients with forearm shaft fracture

Between April 2010 and November 2018, forearm shaft fractures of a total of 300 pediatric patients were treated at the level one pediatric trauma center, of whom 81 patients were excluded either due to surgery at another trauma center, polytrauma or a missing postoperative in-house follow-up at week 4 (Fig. 1). Among the 219 patients analyzed, 128 (= 58.4%) were male and 91 (= 41.6%) were female with a median age of 7 years (IQR 5–9) at the time of fracture. In 118 (53.9%) patients, the left and in 100 (45.7%) patients the right forearm was fractured. One patient sustained a bilateral forearm fracture. Tissue damage was assessed according to the Tscherne and Oestern classification for either closed or open fractures [14]. Of the 209 patients who experienced a closed fracture, 97 (44.3%) had no soft tissue damage, 94 (42.9%) had a first-degree soft tissue damage and 17 (7.8%) had second-degree soft tissue damage. Among the remaining 11 patients who sustained an open fracture, 9 patients had first-degree soft tissue damage and 2 patients had second-degree soft tissue damage. At the time of admission, most patients (92.7%) suffered neither from concomitant injuries nor from injuries at other body sites. 5 children reported paresthesia of the affected arm. Additional fractures occurred in 5 patients. Three of these patients had a supracondylar humeral fracture in addition to the forearm shaft fracture, classified as type 13-M/3.1 II according to the AO classification or type II according to the von Laer or Gartland classification [15]. One child had a fracture of the medial epicondyle. One patient had an additional non-displaced fracture of the olecranon, but this was only detected in the second postoperative period (Table 2).

**Table 2:** Clinical characteristics of pediatric patients with forearm shaft fractures#

Variable	Total Cohort (n = 219)
Median age – yrs. (IQR)	7 (5–9)
Sex – no. (*%*)	
Male	128 (58.4)
Female	91 (41.6)
Fracture side (*%*)	
Right	100 (45.7)
Left	108 (53.9)
Both sides	1 (0.5)
Soft tissue damage^+§^ (*%*)	
Grade 0^+^	97 (44.3)
Grade 1^+§^	94 (42.9), 9 (4.1)
Grade 2^+§^	17 (7.8), 2 (0.9)
Associated injury (%)	
None	203 (92.7)
Paresthesia	5 (2.3)
Additional fracture	5 (2.3)
Supracondylar fracture	3 (1.4)
Medial epicondyle fracture	1 (0.5)
Olecranon fracture	1 (0.5)
Skin abrasion	2 (0.9)
Cerebral concussion	2 (0.9)
Piercing of the bone	1 (0.5)
USP Fracture	1 (0.5)
ICS	1 (0.5)

*USP* ulna styloid process, *ICS* impending compartment syndrome

^#^Values shown are values at presentation unless otherwise specified

+ Tscherne classification for closed fractures

^§^Tscherne classification for open fractures

### Aberrant radiographic findings and non-radiographic deviations

In total, 12 (5.5%) of the 219 patients had aberrant radiographic findings (ARFs) in the second routinely performed follow-up 4 weeks after surgery. These ARFs included missing or reduced callus formation, re-fracture, loss of reduction, implant loosening, implant migration or unintended plastic implant deformation (Fig. 2). As part of the follow-up, additional (1) pathologies in the physical exam and (2) irregularities in the medical history were documented. Both categories together are referred to as “non-radiographic deviations” (nRD) for simplicity. These nRDs are shown in Table 3. Some patients presented various nRDs. A nRD was documented in a total of 96 (43.9%) patients.

**Table 3:** Radiographic pathological findings at the 4-week follow-up#

Variable	Patients without radio-graphic aberrations (n = 207)	Patients with radiographic aberrations (n = 12)	Total (n = 219)
Patients			
Without nRD (*%*)	122 (58.9)	1 (8.3)	123 (56.2)
With nRD (*%*)	85 (41.1)	11 (91.7)	96 (43.9)
Medical history^!^			
Without pathological findings (*%*)	157 (75.8)	3 (25.0)	160 (73.1)
With pathological findings (*%*)	50 (24.2)	9 (75.0)	59 (26.9)
Pre-existing condition^++^	8	3	11
Refracture^#^	16	3	19
Open fracture	14	2	16
Open reduction	13	4	17
Postoperative fall	6	4	10
Physical exam^§!^			
Without pathological findings (*%*)	161 (77.8)	7 (58.3)	168 (76.7)
With pathological findings (*%*)	46 (22.2)	5 (41.7)	51 (23.3)
Sensorimotor deficits	6	0	6
Wound healing disorders	2	1	3
Soft tissue irritation	4	2	6
Loss of motion	20	1	21
Tenderness to palpation	18	3	21

^#^Values shown are values at presentation unless otherwise specified

+  + Pre-existing conditions with potential effect on bone metabolism

#ESIN osteosynthesis of a refracture, §Physical exam at the 4-week follow-up, !multiple representations of individual patients in sub-categories are permitted

Of the 207 patients without ARFs, a total of 85 (41.1%) patients had nRDs. Again, 11 of the 12 (91.7%) patients with ARFs also had nRDs. Of these, 9 (75%) patients had an irregularity in their medical history. With four events each, open reduction and postoperative falls were the most common. Pathologies in the physical exam were documented in five (41.7%) patients with tenderness to palpation being the most frequent (Table 3).

### Aberrant radiographic findings are more likely in patients with non-radiographic deviations

Of the 96 patients with nRDs, 11 (11.5%) had ARFs. In the remaining 85 (88.5%) this attribute was not identified. The group without documented nRDs accounted for 123 Patients, of whom only 1 (0.8%) was found to have an ARF. 122 (99.2%) patients in the group did not have an ARF (Table 3). Pearson’s chi-square showed that the probability of having an ARF differs significantly (p = 0.001) in groups with nRFs and without nRFs in the follow-up 4 weeks after surgery.

### Non-radiographic deviations correlate positively with aberrant radiographic findings

A binary logistic regression was calculated to test the extent to which the factor nRF contributes to the presence of an ARF in the second follow-up. The demographic control variables “gender” and “age” were included. The regression model was statistically significant (X^2^(3) = 17.861 p < 0.001) with an acceptable variance (Nagelkerkes R^2^, R^2^ = 0.226). A high goodness of fit was proven (Hosmer–Lemeshow test, X^2^(8) = 5.116, p > 0.05). Of the three variables included in the model, the variable nRD (p = 0.012) exerted a statistically significant influence on the occurrence of an ARF in the follow-up 4 weeks after surgery. Sex (p = 0.266) and age (p = 0.083) did not have a statistically significant effect. Once a child has a nRD, the probability of an ARF in the follow-up 4 weeks after surgery increases by a factor of 14.554 (95% confidence interval (CI) [1.823–116.163]) (Table 4).

**Table 4:** Statistical relationship of variables#

Variable	Regression coefficient b	SE	Wald test	Sig	OR	CI
Patients						
nRD	2.678	1.060	6.384	0.012	14.553	1.823 – 116.163
Sex	0.785	0.705	1.240	0.266	2.193	0.550 – 8.735
Age at time of fracture	0.169	0.098	3.015	0.083	1.185	0.978 – 1.434

*nRD* non-radiographic deviation, *SE* standard error, *Sig.* significance, *OR* odds ratio, *CI* confidence interval

^#^Values shown are values at presentation unless otherwise specified

### Only irregularities in the medical history indicate aberrant radiographic findings

In order to determine the influence of the nRD subgroups, (1) pathologies in the physical exam and (2) irregularities in the medical history, on the presence of an ARF, statistical calculation was performed according to the previous caption (*Caption 3.2.2*) Of the four variables included in the model, only the variable “irregularities in the medical history” (p = 0.003) showed a statistically significant influence, while the variables “pathologies in the physical exam” (p = 0.193) but also “gender” (p = 0.403) and “age at fracture” (p = 0.141) had no significant influence on the predictive power of the model. As soon as a child has a documented irregularity in the medical history, the chance of an ARF increases by a factor of 8.134 (95% CI [2.061–32.104], Table 5).

**Table 5:** Statistical Relationship of Variables (Subgroups) #

Variable	Regression coefficient b	SE	Wald test	Sig	OR	CI
Patients						
nRD						
Medical history	2.096	0.7	8.955	0.003	8.134	2.061 – 32.104
Physical exam	0.855	0.657	1.695	0.193	2.352	0.649 – 8.524
Sex	0.599	0.715	0.7	0.403	1.820	0.448 – 7.395
Age at time of facture	0.149	0.101	2.168	0.141	1.160	0.952 – 1.413

*nRD* non-radiographic deviation, *SE* standard error, *Sig.* significance, *OR* Odds ratio, *CI* confidence interval

^#^Values shown are values at presentation unless otherwise specified

### Changes in the postoperative protocol are more common in pediatric patients with non-radiographic deviations

Of the 219 pediatric patients, 26 children (11.87%) had a change in the postoperative protocol after the second follow-up. In 193 patients (88.13%) no changes have been made. In total, nRDs were documented in 96 patients (43.8%). In this group, there was no change in 72 children (75.0%) after the second follow-up and in 24 children (25.0%) there was a change in the standard after the second follow-up. nRDs were documented in 123 children (56.16%). In this group, there was no change in 121 patients (98.37%) and a change in the postoperative protocol in 2 cases (1.62%). Pearson’s chi-square revealed that the probability of changing the standard procedure after the 2nd follow-up differed statistically significantly (p = 0.001) in both groups (Table 6).

**Table 6:** Change in postoperative protocol after second follow-up#

Variable	No change in postoperative protocol (n = 193)	Change in postoperative protocol (n = 26)	Total (n = 219)
Patients			
Without nRD (*%*)	121 (62.7)	2 (7.7)	123 (56.2)
With nRD (*%*)	72 (37.3)	24 (92.3)	96 (43.8)
Medical history^!^			
Without pathological findings (*%*)	151 (78.2)	9 (34.6)	160 (73.1)
With pathological findings (*%*)	42 (21.8)	17 (65.4)	59 (26.9)
Pre-existing condition^++^	7	4	11
Refracture^#^	15	4	19
Open fracture	11	5	16
Open reduction	12	5	17
Postoperative fall	3	7	10
Physical exam^§!^			
Without pathological findings (*%*)	154 (79.8)	14 (53.8)	168 (76.7)
With pathological findings (*%*)	39 (20.2)	12 (46.2)	51 (23.3)
Sensorimotor deficits	5	1	6
Wound healing disorders	0	3	3
Soft tissue irritation	3	3	6
Loss of motion	17	4	21
Tenderness to palpation	17	4	21

^#^Values shown are values at presentation unless otherwise specified

### Non-radiographic deviations correlate positively with changes in the postoperative protocol

A binary logistic regression was also calculated to determine the extent to which the “nRD” contributes to the change from the standard postoperative protocol in the 2nd follow-up examination. The demographic control variables “gender” and “age” were also included in the model. The regression model was statistically significant, χ^2^(3) = 32.188 p < 0.001, with an acceptable variance explained by Nagelkerke’s R^2^ = 0.264. Of the three variables included in the model, only the variable “nRD” (p < 0.001) showed a statistically significant impact on the change in the postoperative protocol, while the variables “gender” (p = 0.354) and “age” (p = 0.741) had no statistically significant impact on the predictive performance of the model. If the child has a “nRD”, the chance of a change in the protocol increases after the 2nd follow-up with an odds of 19.297 (95% CI [4.41–84.36]) (Table 7).

**Table 7:** Statistical relationship of variables#

Variable	Regression coefficient b	SE	Wald test	Sig	OR	CI
Patients						
nRD	2.960	0.753	15.467	0.000	19.297	4.414 – 84.363
Sex	0.064	0.069	0.859	0.354	1.066	0.931 – 1.221
Age at time of fracture	0.152	0.461	0.109	0.741	1.165	0.472 – 2.873

^#^Values shown are values at presentation unless otherwise specified

### Pathologies in the physical exam and irregularities in the medical history may lead to a change in the postoperative protocol

Again, a binary logistic regression was calculated to examine the extent to which the nRD subgroups (1) pathologies in the physical exam and (2) irregularities in the medical history contribute to a change in the standardized postoperative protocol after the second follow-up at week 4 (*Caption 2.3*). The demographic variables "gender" and "age" were included in the model. Of the four variables included in the model, both the variable (1) pathologies in the physical exam (p = 0.004) and (2) irregularities in the medical history (p < 0.001) were statistically significant, while other variables did not have significant effect (Table 8).

**Table 8:** Statistical relationship of variables (Subgroups)#

Variable	Regression coefficient b	SE	Wald test	Sig	OR	CI
Patients						
nRD						
Medical history	1.968	0.474	17.273	0.000	7.157	2.829 – 18.107
Physical exam	1.357	0,476	8.121	0.004	3.883	1.527 – 9.872
Sex	0.003	0.467	0.000	0.995	1.003	0.402 – 2.505
Age at time of fracture	0.49	0.071	0.467	0.494	1.050	0.913 – 1.207

*nRD* non-radiographic deviation, *SE* standard error, *Sig.* significance, *OR* odds ratio, *CI* confidence interval

^#^Values shown are values at presentation unless otherwise specified

## Discussion

The radiation exposure from a single conventional imaging examination is relatively low. However, it must be emphasized that frequent imaging not only contributes to the cumulative radiation dose and thus increases the individual radiation risk, but also the collective radiation risk of an entire population. This is particularly important for children, who are especially susceptible to radiation due to their long life expectancy and their particular tissue physiology. Avoiding redundant radiographs after forearm ESIN osteosynthesis, one of the most common surgical procedures on the developing skeleton, could make a significant contribution to improving radiation hygiene [16–18]. This monocentric, retrospective study tries to challenges the concept of standardized and undifferentiated radiological follow-ups by investigating the clinical benefit of radiological follow-up 4 weeks after ESIN osteosynthesis in pediatric diaphyseal forearm fractures.

The study concludes that routinely performed postoperative X-ray follow-ups do not provide additional information for postoperative management if the disease course is otherwise uneventful. Notably, of the 123 patients without nRDs, only one was found to have an ARD (absolute risk (AR) = 0.008). ARDs only occurred more frequently when additional nRDs were detected (OR 14.553, p = 0.012). In this group, irregularities in the medical history were particularly associated with the occurrence of ARDs (p = 0.003). This confirms the overriding importance of a complete medical history and thorough physical examination, to which radiological follow-up examinations should be subordinate and indicated only in the presence of nRDs. This conclusion is in line with the findings of Oehme et al., who in a monocentric, prospective study (n = 316) were able to rule out any improvement in patient care from early postoperative radiographic follow-up of adult ankle- and distal radius fractures as long as the clinical course was normal [11]. For this group of patients, a first X-ray follow-up 6 weeks postoperatively, the time of sufficient bony consolidation, was recommended [11]. Due to the even smaller number of cases of bony non-union after ESIN osteosynthesis in pediatric forearm shaft fractures described in the literature, which mostly occurred after open fracture or open reduction, the interval until the first X-ray follow-up could be extended to the intended time of implant removal, six months postoperatively [19, 20]. Interestingly, Oehme et al. also showed that the absence of an early standardized postoperative X-ray follow-up had also no negative effect on the quality of care as perceived by the patient [11]. The implementation of safe and cost-effective fracture ultrasound could nevertheless provide additional reassurance for the examiner and the patient during the post-operative interval. This diagnostic tool is particularly suitable for the follow-up of fractures of the long bones and is significantly more sensitive in detecting the formation of callus than conventional X-ray imaging [21, 22]. Based on these studies, it can be assumed that a more cost-conscious follow-up protocol with a lower radiation exposure is both necessary and feasible as long as the course of the disease is uneventful.

Due to the monocentric and retrospective design of the study, there is an increased risk of selection bias, which could have an impact on the generalizability of the results. Although the follow-up rate is relatively high at 80%, the proportion of patients who withdrew from the 4-week follow-up at our center may also have led to a selection bias. In some cases, these may have been patients who continued their treatment at a different clinic due to a complication of their treatment. However, it seems more likely that follow-up controls were carried out by specialists in topographical proximity to the patient’s home. This is consistent with the high number of material removals that we performed after the end of the 6th postoperative month. Of the 300 patients treated with forearm ESIN osteosynthesis during the study period, 277 (92.34%) underwent implant removal at our center.

## Conclusion

This study provides strong indications that a routine X-ray follow-up four weeks after ESIN osteosynthesis does not enhance patient care in the absence of any other complications. A more cautious use of X-ray diagnostics in favor of a detailed medical history and clinical examination could increase patient safety in the postoperative management of forearm shaft fractures.

## Data Availability

No datasets were generated or analysed during the current study.

## Abbreviations

AR: Absolute risk
ARF: Aberrant radiographic finding
CI: Confidence interval
ESIN: Elastic stable intramedullary nailing
ICS: Impending compartment syndrome
IQR: Interquartile range
nRD: Non-radiographic finding
OR: Odds ratio
SE: Standard error
Sig: Significance
USP: Ulna styloid process
